# The Etiology of Chronic Nephritis

**Published:** 1902-08-16

**Authors:** 


### The Etiology of Chronic Nephritis.

The etiology of chronic nephritis is very obscure. That it arises in many cases in association with the gouty process is sufficiently obvious, but unfortunately the origin of gout itself is a matter still much in dispute. It seems, however, to be pretty generally admitted that the toxin by whose injurious action that widespread degeneration which goes by the name of chronic Bright's disease is set up, is due to some perversion or imperfection of the metabolism within the tissues.

Dr. A. E. Elliot, of Chicago, however, speaking at the recent meeting of the American Medical Association, has declared his belief that the absorption of toxic material from the intestinal tract plays the most important part in its etiology. As a curious and interesting example of the manner in which the same facts may be differently interpreted according to the light in which they are regarded we would refer to what Dr. Elliot says concerning the relation between kidney disease on the one hand and diet and purgation on the other. In the treatment of chronic kidney disease it has long been held by many observers that a careful dietary is a matter of the first importance. Of late some earnest protests have been made against the views which were generally held on the subject, but certainly until quite recent times the aim and intent of most practitioners in devising a dietary for a case of chronic Bright's disease has been to provide one the waste products resulting from the digestion of which should not take the form of substances likely to irritate the kidneys or embarrass them in the performance of their functions. Again, the relief given to the nephritic symptoms by purgatives has been regarded as analogous with the relief often given by Turkish baths, due in both cases to a removal of the toxins from the blood, in the one case by the intestines and in the other by way of the skin.

To Dr. Elliot, however, these facts appear in a very different light. Considering that, as in the nephritis caused by plumbism so in the other cases, the kidney disease is due to the presence in the blood of some irritating toxic material, and looking as he does to decomposition of the intestinal contents as the source and perennial fount from which this toxin is absorbed, he regards careful dieting as advantageous, because by its means—by giving a food little likely to decompose—the supply of toxins may be diminished; while he regards the morning purge, the clearing out of the colon, and the excitation of the functions of the intestine which are admitted to do so much good, as doing it, not by increasing the elimination of waste products circulating in the blood, but by clearing away the very source from which the blood is becoming poisoned.

It is an easy thing, no doubt, to assume that the intestinal contents which are the source of all good—the pabulum from which the tissues derive their nutriment, the muscles their strength, the very brain its power of thought—are also the root of all evil, and among other evils, of chronic nephritis; yet the theory would be more satisfactory could we lay our fingers on the poison. Still there is much to be said for Dr. Elliot's contention, and there certainly is this great thing to make one cling to it, namely, that if true it throws a far more hopeful light upon the treatment of this disease, if caught at an early stage, than seems to be cast by other and more generally accepted views as to its etiology. Dr. Elliot points out, as emphasising his text, that when the kidneys fail in their excretory function, the liver cells degenerate because of the toxic substances which are present in the blood; that the liver then fails to metabolise substances which come to it, and adds its own quota of toxic material to the blood, thus still further irritating the kidney, and that the vicious circle thus formed continually deteriorates the general condition of the patient.

The relation of chronic nephritis to mode of life seems to have a distinct bearing upon this problem, for the inactive life led by so many city people, people who are more especially the victims of this disease, undoubtedly tends to promote those conditions in which toxæmia from intestinal decomposition would be most likely to occur. These people almost invariably over-eat themselves, and as showing that the result is to throw upon the kidney the excretion of toxins likely to be injurious to its tissues, Dr. Elliot points out that in such people the urine is often quite toxic when injected into animals. The basis of these metabolic disturbances is often, he says, an atonic intestinal catarrh. And perhaps it may be so. That ultimately the vicious circle above alluded to is set up, and that in many cases before the patient has come under treatment, his disease has led to degenerations which it is beyond the power of medicine to cure, is true enough. But it may be that in origin, long before the development of symptoms, the disease had its commencement in some form of toxæmia starting in the intestinal contents; a much simpler affair than that inborn tendency to perverted metabolism to which some have recourse in explaining the etiology of nephritis.

